# Community Health Worker Perspectives on Engaging Unhoused Peer Ambassadors for COVID-19 Vaccine Outreach in Homeless Encampments and Shelters

**DOI:** 10.1007/s11606-022-07563-9

**Published:** 2022-04-11

**Authors:** Kristen Choi, Ruby Romero, Priyanka Guha, Gunner Sixx, Allison D. Rosen, Ashley Frederes, Jacqueline Beltran, Julissa Alvarado, Brooke Robie, Lindsey Richard, Anthony Coleman, Adam Rice, Marisol Rosales, Angel Baez, Emily Thomas, Chelsea L. Shover

**Affiliations:** 1 UCLA School of Nursing, Los Angeles, CA USA; 2 Department of Health Policy and Management, UCLA Fielding School of Public Health, Los Angeles, CA USA; 3 Division of General Internal Medicine and Health Services Research, Department of Medicine, UCLA David Geffen School of Medicine, Los Angeles, CA USA; 4 Housing for Health, Los Angeles County Department of Health Services, Los Angeles, CA USA; 5 Department of Epidemiology, UCLA Fielding School of Public Health, Los Angeles, CA USA; 6 Department of Community Health Sciences, UCLA Fielding School of Public Health, Los Angeles, CA USA; 7 Los Angeles Community Action Network, Los Angeles, CA USA

## Abstract

**Background:**

COVID-19 vaccination is a priority for people experiencing homelessness. However, there are barriers to vaccine access driven in part by mistrust towards clinicians and healthcare. Community health workers (CHWs) and Peer Ambassadors (PAs) may be able to overcome mistrust in COVID-19 vaccine outreach. An unhoused PA program for COVID-19 vaccine outreach by CHWs was implemented in Los Angeles using a participatory academic-community partnership.

**Objective:**

The purpose of this study was to evaluate CHW perspectives on an unhoused PA COVID-19 vaccine outreach program in Los Angeles.

**Design:**

This study used a participatory community conference and qualitative focus groups to understand CHW perspectives on the PA program. The one-day conference was held in November 2021.

**Participants:**

Of the 42 conference participants, 19 CHWs participated in focus groups for two-way knowledge exchange between CHWs and researchers.

**Approach:**

Four focus groups were held during the conference, with 4-6 CHWs per group. Each group had a facilitator and two notetakers. Focus group notes were then analyzed using content analysis to derive categories of findings. CHWs reviewed the qualitative analysis to ensure that findings represented their experiences with the PA program.

**Key Results:**

The five categories of findings from focus groups were as follows: (1) PAs were effective liaisons to their peers to promote COVID-19 vaccines; (2) CHWs recognized the importance of establishing genuine trust and equitable working relationships within CHW/PA teams; (3) there were tradeoffs of integrating unhoused PAs into the existing CHW workflow; (4) CHWs had initial misgivings about the research process; and (5) there were lingering questions about the ethics of “exploiting” the invaluable trust unhoused PAs have with unhoused communities.

**Conclusions:**

CHWs were in a unique position to empower unhoused PAs to take a leadership role in reaching their peers with COVID-19 vaccines and advocate for long-term employment and housing needs.

## INTRODUCTION

People experiencing homelessness have been disproportionately harmed by the COVID-19 pandemic as a result of inequities in pandemic response. COVID-19 case fatality rates are higher for people experiencing homelessness compared to their housed counterparts.^1^ Studies of COVID-19 in homeless shelters have found high levels of COVID-19, with higher levels among those living communally or in crowded settings.^2–4^ People experiencing homelessness have few options for handwashing, accessing masks, social distancing, and accessing COVID-19 information.^5,6^ They may also have a harder time accessing COVID-19 tests and vaccines due to costs, lack of insurance, lack of transportation, and negative prior experiences with health systems.

Evidence suggests that people experiencing homelessness are interested in vaccination, but that 30–50% of people experiencing homelessness have concerns or questions about vaccines.^7,8^ Overall vaccination levels in the homeless community lag an estimated 15–25% behind levels observed in the population generally.^9^ One study in Los Angeles found that 58% of young adults experiencing homelessness reported that they had not been offered a vaccine, suggesting a further problem of inadequate vaccine access and outreach.^10^ Active vaccine outreach is especially important among people experiencing homelessness because of the long history of marginalization, mistreatment, stigma, and substandard care this population has experienced from healthcare providers.^11–13^

It is important to develop models of COVID-19 vaccine outreach that address issues of access and mistrust, rather than assuming that people experiencing homelessness can easily access existing modalities of vaccine delivery (e.g., mass vaccination sites, pharmacies). Two potential such models are Community Health Worker (CHWs) and Peer Ambassador (PA) models. CHWs are individuals who provide basic health services and health education in low-resource areas, operating directly in the community outside of traditional health systems^14^. They are often purposefully chosen from communities where they will be working to reflect community values, culture, and language and engender trust.^14^ Extending the emphasis on community trust in health delivery models further, PAs are individuals who themselves are members of the target population and can offer peer support and shared experience in navigating health and healthcare.^15^ Peer engagement has been studied in social networks research and peer-based interventions for youth experiencing homelessness, but there is little empirical evidence on the utility of embedding unhoused peers in professional street outreach teams and best practices for such PA integration.^16,17^

Given evidence on the successes of CHWs in reaching marginalized communities with health services and the potential for even greater success with the addition of PAs, the Housing for Health Division of the Los Angeles County Department of Health Services partnered with a local academic institution, the University of California Los Angeles (UCLA), to redesign its street-based COVID-19 vaccine outreach to people experiencing homelessness on principles of trust and peer support. In 2020 and early 2021, Housing for Health operated mobile COVID-19 response teams comprised of nurses, EMTs, and CHWs to offer COVID-19 tests and vaccinations to people experiencing homelessness. In the summer of 2021, Housing for Health partnered with UCLA to embed PAs in their outreach teams, recruiting individuals who had lived experience of homelessness and who had received a COVID-19 vaccine.

### CHW-PA Model of COVID-19 Vaccine Outreach

Additional details about the design and implementation of the PA model are reported elsewhere.^18^ Briefly, each of the COVID-19 response teams operated in one of eight geographic regions of Los Angeles County. Teams would select a homeless encampment or shelter in which to conduct vaccine outreach in their assigned region. CHWs would work in pairs with PAs to first conduct general outreach, including introducing themselves to potential clients; distributing food, water, and harm reduction supplies (e.g., naloxone, clean needles); and assessing potential interest in a COVID-19 vaccine. CHWs were non-clinical health workers selected to reflect the community (i.e., prior lived experience of homelessness or experience working in homeless services) and provide basic health resources. If clients were receptive to receiving a vaccine, CHW/PA pairs would then introduce a clinical team member to provide vaccine education, administer vaccines, or provide other medical care (e.g., wound care, blood pressure checks, COVID-19 testing, referrals to clinics). Clients could be recruited to the PA program after completing the COVID-19 vaccination series, as could others who were vaccinated that CHW/PA pairs met during the outreach process. PAs could work up to 20 h with COVID-19 response teams and were paid $25 an hour in gift cards (Kroger or Target).

### Study Purpose

The PA model used by Housing for Health and UCLA for COVID-19 vaccine outreach was implemented and evaluated using a community-partnered participatory research approach.^19^ Community-partnered participatory research is an approach that emphasizes equal partnership between academic researchers and community members, shared power, shared ownership of research projects, and broader coalition-building for community action in research.^19–21^ As part of the overall PA program evaluation, we held a participatory community conference to explore perspectives on the strengths and limitations of the PA program from the vantage point of CHWs, who were largely responsible for program implementation and data collection. Given the newness of this CHW-PA model for COVID-19 vaccine outreach among people experiencing homelessness, understanding how CHWs view the model has potential to inform model refinement and application of this model to other areas of health. This article reports findings from the community conference, designed to engage CHWs in evaluating and refining a PA model for COVID-19 vaccine outreach in homeless encampments and shelters in Los Angeles.

## METHODS

### Participatory Community Conference Approach

This project used a participatory community conference to explore the PA model from the perspective of CHWs, who had the greatest level of interaction with PAs. As a quality improvement initiative, this project was determined by the UCA IRB to be exempt from IRB oversight. Community conferences are a methodology for engaging community members in participatory research.^22^ A community conference is a gathering of community members, scientists, direct service providers, and other relevant research stakeholders to engage a community in the process of research, facilitate two-way knowledge exchange, and ensure equitable, shared ownership of a research project and its outcomes.^22,23^ This approach arose from community-based organizations as a response to longstanding untrustworthiness, unequal power, lack of community voice, and lack of tangible action that was and still is pervasive in scientific research with minoritized communities.^19,22^ Community conferences differ from traditional academic or professional conferences in several ways. A community conference relies on two-way knowledge exchange rather than one-way didactic presentations with passive participation. It is intentionally designed to increase community voice, improve use of community resources, and decrease distrust between community members and researchers. Finally, community conferences are designed to inform utilization of research findings for action in a community, going beyond scientific dissemination in an academic journal alone.^23^

### Participants and Setting

The one-day conference took place at UCLA. Conference attendance was free and participants were provided with lunch, refreshments, parking, and small conference favors. Participants included CHWs, research staff and faculty, and Housing for Health education program managers and coordinators, for a total of 42 conference participants. Of these conference attendees, there were 19 CHWs who had been involved in the PA COVID-19 vaccine outreach program who participated in focus groups. We did not include other members of outreach teams (e.g., PAs, nurses) in the conference or focus groups to promote honest reflection and open discussion about the model.

### Focus Groups

During the conference, focus groups were used to facilitate two-way knowledge exchange between CHWs and researchers.^22,23^ Each group had four to six CHWs, two note-takers, and a facilitator. The facilitators and note-takers were research team staff members and Housing for Health education team staff with a bachelor’s or master’s degree (authors R. R., P. G., G. S., A. R., A. F., J. C., B. R., L. R., C. D.). The groups lasted approximately 45 min and followed a semi-structured interview guide to explore CHW perspectives on program successes, challenges, and approaches for improving the PA model. Focus groups were not recorded to promote honest dialogue and to protect participant anonymity, but there were detailed notes recorded for each focus group (four total focus groups) that were used for analysis.

### Data Analysis

Content analysis was used to organize focus group notes into categories of key findings.^24,25^ We first reviewed focus group notes and coded the data inductively, using gerund coding as a first step to center the action of participants.^26^ Gerund coding, also called process coding, involves exclusively using gerunds (–ing words) to code data to orient coders towards considering the action of participants, not just topics appearing in participant statements.^26^ Following initial gerund coding, codes were then organized into categories and subcategories of similar findings. We used an iterative, inductive process to group similar codes that remained reflective of the action of participants. There were 30 initial codes, which were reflective of nine subcategories and five overall categories of findings.

The initial coding was performed by one author with a PhD and background in qualitative research methods (KC), then reviewed and validated with the rest of the authorship team in two coding meetings. Based on the code review and validation, codes were added and modified until all authors were in agreement that codes adequately represented the data. The same process of review/validation was repeated to derive categories and subcategories of findings. Four CHWs who participated in focus groups reviewed and revised the coding, subcategories, and categories to ensure that they reflected perspectives of CHWs.

## RESULTS

A total of 19 CHWs participated in four focus groups. The five categories of findings from these focus groups were as follows: (1) PAs were highly effective liaisons to their peers to promote COVID-19 vaccine uptake; (2) CHWs recognized the importance of establishing genuine trust and equitable working relationships within CHW/PA teams for effective outreach to occur; (3) there were tradeoffs of integrating unhoused PAs into the existing CHW workflow; (4) CHWs had initial misgivings about integrating the research process into their existing workflow; and (5) there were lingering questions about the ethics of “exploiting” the invaluable trust unhoused PAs have with unhoused communities.

### PAs Were Highly Effective Liaisons to Their Peers to Promote COVID-19 Vaccine Uptake

#### Subtheme: CHWs Discovered the Value of Accepting PA-Led Approaches to Outreach as Members of the Unhoused Community

CHW participants saw great value in tapping into the preexisting rapport and trust PAs had with their unhoused neighbors based on a shared experience of homelessness. PAs were universally viewed by CHWs as the best vaccine “salesmen” within the outreach teams. They felt that because PAs were knowledgeable about the community, had lived experience of homelessness, knew the best language to use to discuss vaccines, and were respected by their neighbors, they were able to be successful in vaccine outreach. CHWs recognized that they needed to follow the lead of PAs in the best way to reach out to a community, even if it was unfamiliar to the CHW. A CHW emphasized, “Let them take the lead. They know their community.” Another CHW stated, “Let them be themselves when they are speaking, letting them use their own slang or dialect even though they are talking about something very serious.”

#### Subtheme: CHWs Learned How to Identify the Most Effective PAs to Integrate with Their Teams

Along with preexisting trust and shared experience of homelessness, CHWs felt that PAs with strong interpersonal skills and who were genuine vaccine advocates were most successful in reaching their peers. They valued PAs who were calm, kind, non-judgmental, and confident; who were not easily offended; and who knew the community (e.g., “popular”). One CHW said, “A good way to identify PAs is when you see someone taking care of the encampment/environment they are located in. It shows someone that cares for the community.” While CHWs recognized that some PAs were likely motivated to participate in the program for the gift card incentives, they noted that those who also had “passion” and felt “positive about the vaccine” were ideal PAs who were effective at reaching their peers.

### CHWs Recognized the Importance of Establishing Genuine Trust and Equitable Working Relationships Within CHW/PA Teams for Effective Outreach to Occur

CHWs felt that it was of high importance to integrate PAs into the outreach teams and ensure that they felt supported, included, and empowered. They emphasized the importance of framing PA involvement as “working alongside you, not for you,” as well as treating PAs “like colleagues and valu[ing] their knowledge.” CHWs felt that it was their responsibility to set PAs up for success by giving them opportunities to tell their vaccine story, affirming that PAs were part of a team and had the full backing of the entire outreach team including medical personnel, and introducing PAs as colleagues during outreach attempts. One CHW described a process of “hold[ing] space” for PAs at the very beginning of outreach events for PAs to take the lead in educating the team on the encampment or area, giving suggestions for outreach, and meeting the clinical team.

As a result of these efforts to promote equitable PA involvement with the outreach team, there was substantial and sometimes surprising bidirectional learning that CHWs experienced while working with the PA program. CHWs saw themselves as having a “mentor relationship” to PAs. One CHW said of their role, “It’s like a stepping stone for them to see what they might like to do in the future. [I] feel like I’m being purposeful.” CHWs reflected on learning to take a less pressured, volume-driven approach to their vaccination work, improving their communication skills, and discovering how to empower people to be leaders within their own communities. They also noted the humanizing nature of a collaborative approach and the value of being reminded that “the clients we serve are just like us.”

### There Were Tradeoffs of Integrating Unhoused PAs into the Existing CHW Workflow

CHWs were positive about the PA program as a whole, but noted that there were costs and tradeoffs to integrating PAs into their existing COVID-19 vaccine outreach program. One example of program tradeoffs was new safety concerns. The program necessitated that CHWs carry gift cards and phones, which might make them targets for theft. They also talked about situations where PAs had preexisting conflicts with encampment communities (e.g., past violent encounters, owing people money) and that they generally knew little about the backgrounds of the PAs who were working with their teams. Involving PAs also had restricting effects on their outreach efforts in some cases, both geographically and in terms of outreach volume. For example, one focus group participant noted, “When a PA does not want to leave their area or their belongings it can limit PA participation or put their belongings at risk in their absence.” They talked about PA integration as “growing pains,” which sometimes felt “like pulling teeth and it slows things down.” CHWs felt pressure to recruit new PAs and supervise PAs while continuing to do their usual volume and quality of vaccine outreach, which, in the words of one participant, “sometimes feels like we’re doing multiple people’s jobs at the same time.”

There were several barriers to optimal, full participation of PAs in vaccine outreach noted by CHWs. Communication was a challenge noted by all focus groups. They gave examples of when they could not contact PAs because they had moved, run out of phone minutes, or had their phones lost/stolen. Program retention was also a challenge, as PAs sometimes changed their minds about participation or could not participate due to unforeseen circumstances. One note from a participant comment stated, “People are excited and agree but then don’t follow through – [I] don’t want to make them feel pressured to be a PA but a lot of time is spent recruiting them.” CHWs felt strongly that mobile teams did not work well with PAs, as PAs may not necessarily know or have trust with encampments outside their own and may be unable or unwilling to leave their belongings. Another barrier to mobile teams was transportation; CHWs were unable to transport PAs in Los Angeles County vehicles, which made moving between outreach sites difficult or impossible and was detrimental to the “sense of belonging and inclusiveness in the team,” in the words of one participant. Finally, encampment sweeps by city sanitation workers were perceived to interfere substantially with both PA participation and overall vaccination efforts.

### CHWs Had Initial Misgivings About Integrating the Research Process into Their Existing Workflow

Because the PA program was implemented as a research study with an academic partnership, the program required training on and implementation of informed consent, tracking of gift card incentives, and collection of evaluation data. The research aspects of the program were initially perceived by CHWs as “annoying bureaucratic shit” and “bureaucratic extra work.” One CHW stated, “I am just not into data. Nothing personal, I just don’t like it. I just pretend.” They found that the consent and tracking aspects of the study felt “like a burden,” including both the paperwork and perceived pressure to recruit PAs. Although the research aspects of the program were initially difficult for CHWs, several ultimately found the research process to be acceptable and even valuable. CHW participants said, “Everyone seems quick and comfortable with [the] consent process. At first people thought it would feel unnatural or take too much time, but that doesn’t seem to be a problem.” Others reflected, “Learning the consenting process has been a good learning experience for me” and “Look at how many people we are making meaningful connections with by the surveys.”

### There Were Lingering Questions About the Ethics of “Exploiting” the Invaluable Trust Unhoused PAs Have with Unhoused Communities

CHWs expressed an acute awareness of power dynamics and potential risk for exploitation at play between themselves as county workers and unhoused PAs. They perceived that recruiting PAs could feel like “tokenizing” an unhoused person and wanted to be cautious not to appear to be “exploiting” people experiencing homelessness. One participant framed this intention as, “We are the people; we aren’t here to exploit the people.” CHWs recognized that some PAs might be motivated by the gift card payments more so than the actual work of vaccine outreach, and that the gift cards may be coercive. On the other hand, CHWs also felt that the gift card program itself was problematic as a payment source and insensitive to the needs of PAs. For example, gift card offerings were sometimes for stores that were too far for PAs to access or stores that were perceived as too expensive. CHWs felt that it was of the utmost importance for them to recognize that PAs are “being vulnerable by allowing you into their space and introducing you to the community” and to “honor that trust by not ever taking advantage of the trust the PA bestows upon you.”

A great source of frustration to CHW participants was their inability to provide more long-term assistance to PAs. They wanted to pay PAs with cash instead of gift cards, give out tents, offer skills training or job opportunities, assist with resumes, and connect PAs to case workers for assistance with securing stable, affordable housing. CHWs also saw potential for longer-term employment of program participants as PAs or even eventually as CHWs. As county employees, CHWs had access to service tracking systems and felt that short-term PA engagement was a missed opportunity to offer long-term employment and housing support.

## DISCUSSION

This community conference held to engage CHWs in the evaluation of a PA model for COVID-19 vaccine outreach revealed the value of a peer approach to vaccination and opportunities to make the program more sustainable and equitable. CHWs perceived PAs to be valued members of outreach teams because of the preexisting trust and shared experience PAs had with unhoused communities. PAs used outreach strategies and language that CHWs could not, making them invaluable team members. In attempting to leverage preexisting trust with potential vaccine recipients, CHWs described the importance of building trusting relationships within the outreach teams.^27^ They were careful to ensure that PAs felt included, accepted, supported, and had opportunities for team leadership. This attention to intra-team dynamics resulted in mentorship opportunities and learning for the CHWs, consistent with other research on peer support models for providing health services within homeless communities.^28–30^

Although nearly all CHWs felt that the PA program was successful, they noted challenges and tradeoffs to this approach. Integrating PAs into outreach teams introduced new safety concerns and at times slowed outreach down. The teams experienced challenges with communication, transportation, PA retention, and navigating encampment sweeps that displaced both PAs and potential vaccine recipients. Another program barrier from the perspective of CHWs was the research process, a phenomenon that has been well-described in the literature on community-partnered participatory research.^19,31–33^ Informed consent, incentive tracking, and surveys required for evaluating the PA model were perceived as cumbersome, bureaucratic, and time-consuming. Some CHWs felt that it introduced too much pressure to recruit PAs with every outreach attempt, which was not always feasible. Although many CHWs came to understand and even appreciate the process of research, this finding highlights the importance of using partnered research approaches with marginalized communities.^19^

The CHWs involved with the PA program were highly sensitive to power dynamics that might exist in their relationships with PAs. They expressed concern about the potential to “exploit” or “coerce” unhoused PAs by relying on their trusted relationships with unhoused communities. In light of this risk, CHWs actively worked to ensure that PAs were not marginalized in their outreach teams, followed the leadership of PAs in approaches to outreach, accepted that there might be dual motivations for participation in the program (gift card incentives, caring about vaccination of their communities), and advocated for the program to add opportunities for longer-term employment and housing support for PAs. This identified need for longer-term PA support provides a roadmap for action in refining the PA model, as similar unhoused PA programs may serve the dual function of conducting outreach to unhoused communities and providing a pathway for employment and housing for PAs themselves.

There are strengths and limitations to this community conference approach for evaluating the peer vaccine outreach model. We engaged a group of CHWs who interfaced most directly with PAs among members of the vaccine outreach teams. CHWs provided a unique perspective on program strengths and challenges that are unlikely to be elicited through traditional quantitative methods of program evaluation or qualitative methods that do not use a participatory framework. Limitations of the study include the single-city nature of the PA program and the focus of the program on street encampments and shelters, which does not capture all forms of homelessness. The study focused only on adults who spoke English or Spanish. This study did not elicit the perspectives of PAs themselves, which is an important knowledge gap and direction for future research. Finally, the sociopolitical climate around homelessness in Los Angeles during the time of the PA program meant that there were relatively frequent encampment sweeps and concerted efforts by the city to dissolve street encampments.^34^ These circumstances may have introduced barriers to PA participation and communication that affected how CHWs viewed the program.

Engaging CHWs in a participatory community conference was a generative approach to evaluating a peer model for COVID-19 vaccine outreach. Future studies should explore the strengths and limitations of a CHW-PA vaccine outreach model from the perspective of PAs themselves to refine the model and ensure that it is acceptable to PA team members. CHWs provided perspective on the strengths and challenges of the program, considerations for how it felt to participate in a research-based implementation of the program, and actions to make the program more equitable and sustainable for PAs. CHWs occupy the unique position of interfacing with the clinical team and Los Angeles County, but also interfacing with unhoused communities. This position allowed CHWs to empower PAs to take a leadership role in reaching their peers with COVID-19 vaccines and realize the value of their trusting relationships and shared experiences of homelessness. There is potential to integrate PAs into existing CHW models of vaccine outreach, as well as to consider CHW-PA models for providing other types of health services in street and encampment settings.
